# Examining Factors That Influence Learner Retention in MOOCs During the COVID-19 Pandemic Time

**DOI:** 10.1177/21582440231175371

**Published:** 2023-05-30

**Authors:** Zhonggen Yu, Liheng Yu

**Affiliations:** 1 Beijing Language and Culture University, Beijing, China; 2 Jiangsu Ocean University, Lianyungang, China; 3 University of Birmingham, Edgbaston Birmingham, UK

**Keywords:** retention, MOOCs, information technology, influencing factors

## Abstract

Massive Open Online Courses have become a frequent platform for learners to acquire knowledge. This study aims to explore multiple factors influencing learner retention in MOOCs during the COVID-19 pandemic. To address this, we collected quantitative and qualitative data from questionnaires and qualitative data from interviews and then analyzed them through the Partial Least Square–Structural Equation Modeling to test 14 research hypotheses. The proposed research model and research hypotheses are empirically tested with 243 participants across the world. According to the results, support is found for all of the 14 research hypotheses. We confirmed 14 factors influencing learner retention in MOOCs. The result is beneficial for designers and manufacturers of MOOCs to improve the quality of the products and facilitate online or blended learning during this special time. It could also help students improve their learning experiences. Future research could examine influencing factors of learner retention in MOOCs with interdisciplinary cooperation.

## Introduction

The term “Massive Open Online Courses (MOOCs)” was coined by Dave Cormier and Bryan Alexander in 2008 (Brahimi & Sarirete, 2015). MOOCs were defined as a platform to present digital educational programs by improving great coverage through open methods (Conole, 2016). MOOCs, originally aiming to provide open and free courses for university students (Yuan & Powell, 2013), were also defined as a method to deliver knowledge through limitless enrolment, where anyone was allowed to join, learning activities were realized through the Internet, and the courses were designed based on given learning goals (Thompson, 2011). As a relatively innovative delivery model, MOOCs aimed to boost the massive engagement of learners (Shapiro et al., 2017). Although COVID-19 did not promote MOOCs, it promoted online teaching techniques for classes that had not been taught online and sharply increased the enrollment and engagement in MOOCs (Pinto et al., 2020). Most of learners (98%) perceived that COVID-19 started the prosperity of digital learning, which promoted the development of online education and virtual educational institutes. This rapid development has stimulated the proliferation of various kinds of applications used for communicative purposes between learners and teachers, among which MOOCs have become a frequent platform for learners to acquire knowledge (Alabdulaziz, 2021).

The new century has been witnessing the popularization of MOOCs (Ruiperez-Valiente et al., 2020). In 2012, top universities in the United States established online learning platforms to provide free courses on the Internet. The rise of Coursera, Udacity, and edX course providers has provided more students with access to systematic online learning. They are all for higher education, and like real universities, they have their learning and management systems. The year 2013 witnessed a dramatic development of MOOCs in Asia. Online courses were offered in Hong Kong University of Science and Technology, Peking University, Tsinghua University, and the Chinese University of Hong Kong (Zhejiang Online, 2013). MOOCs have been rising as a valid teaching approach to reach large populations (Olivares et al., 2021).

It is necessary to study MOOCs during this special pandemic time when we are confronted with educational sustainability (Sosa-Diaz & Fernandez-Sanchez, 2020). However, inadequate studies have explored factors influencing learner retention in MOOCs. To complement the missing link in the literature, this study aims to examine the various factors that may exert a great influence on Learner Retention in MOOC-based learning. It will retrieve plentiful data from students who have experienced MOOC-based learning to identify various influencing factors. The data will be retrieved from both the questionnaires and interviews to understand students’ opinions on the factors that may exert a great influence on their retention of MOOC-based learning behaviors. We will identify the relationships between various influencing factors and Learner Retention through the Partial Least Square–Structural Equation Modeling (PLS-SEM) and semi-structured interviews, as well as students’ responses to the open-ended questions in questionnaires.

## The Statement of the Problem

The purpose of this study is to examine the factors that may exert an influence on Learner Retention since lower Learner Retention has become a serious problem negatively influencing MOOC-based learning outcomes. This study aims to answer the research question: will Learner Retention in MOOCs be correlated with Instructor-to-Learner Interaction (ILI), Learner-to-Learner Interaction, Course Content, Course Structure, Perceived Effectiveness, Instructor Support, Instructor Feedback, Information Delivery, Technology, Quality Resources, The Focus of Subjects, Timing, Flexibility and Scaffolding for Diversity, and Pre-Course Information? The solution to this research question may be beneficial to the reduction of dropout rates and the quality of MOOC-based learning. There are several terms in need of further explanation. Course Content refers to the knowledge in a given discipline available in a course based on MOOCs. The MOOCs platform refers to a virtual or intangible structure, usually constructed through digital technologies, where people log in when they make speeches, give a performance, acquire knowledge, or join learning activities. The size of MOOCs tends to indicate the number of participants who join the MOOCs-based education.

## Literature Review

### Literature on MOOCs During the COVID-19 Pandemic

Since the outbreak of COVID-19, numerous studies have been committed to the use of MOOCs in education during this special time. The COVID-19 pandemic has been witnessing a sharp increase in engagement in MOOCs which has brought real learning experiences to learners and internalized the computational thinking knowledge by assigning peer-graded tasks (Pinto et al., 2020). Computational thinking referred to conceptual skills involving computer sciences and the underlying programming knowledge (Ezeamuzie & Leung, 2021). Emphasis could be placed on the skills of information navigation and analysis in MOOC-assisted learning, as well as the association between learners’ preferred approaches and academic activities through MOOCs (Gonda et al., 2020). When the world is attempting to contain COVID-19, 12 tips have been proposed for medical students to improve the effectiveness of online learning such as MOOCs- or mobile platform-assisted learning, which focuses on instructional approaches, consultation, motivation, ethics, performance evaluation, and revision (Jiang et al., 2021). Besides, this study will examine other factors influencing learner retention in MOOCs during the COVID-19 pandemic.

Learner Retention in MOOCs was previously found under the influence of numerous factors: learner-to-learner interactions, instructor-to-learner interactions (Bernard et al., 2009), Course Content, Course Structure (Yang & Sun, 2018), Perceived Effectiveness (Yang & Sun, 2018), Instructor Support, Instructor Feedback (Barbera et al., 2017), Information Delivery (Fianu et al., 2018), Technology (Alyoussef, 2021), Quality Resources, The Focus of Subjects, Timing (Azevedo & Marques, 2017; Fianu et al., 2018), Flexibility and Scaffolding for Diversity (Hjeltnes & Horgen, 2016), and Pre-Course Information (Alraimi et al., 2015). Therefore, this study will concentrate on the above factors that may influence Learner Retention in MOOCs.

### Interactions in MOOCs

Numerous factors involving learners, instructors, and technologies have been discussed regarding their influence on MOOC-based learning (Ayub et al., 2017). Collaboration and interactions between learners (Riyami et al., 2019), user profile, the experience of making MOOCs, and the level of satisfaction in the interaction with MOOCs strongly influenced Learner Retention in MOOCs (Yamba-Yugsi & Lujan-Mora, 2017). Three interactions, that is, learner-to-learner interactions, instructor-to-learner interactions, and learner-to-content interactions, were considered important factors that might exert a significant influence on Learner Retention in MOOC-based learning (Bernard et al., 2009). The degree of interactions between learners and their similar counterparts could not predict peer interactions (Castellanos-Reyes, 2021). Peer interaction could promote learner engagement in MOOCs (Hew, 2016), possibly leading to improvements in Learner Retention in MOOCs. Learner-to-Learner Interaction was considered indispensable to learner retention in the completion of MOOC-based learning (Tawfik et al., 2017). Instructor-to-Learner Interaction also exerted a great influence on Learner Retention in MOOCs (Bernard et al., 2009). Therefore, we proposed hypotheses as follows:

*H1.* Instructor-to-Learner Interaction is positively correlated with Learner Retention in MOOCs.
*H2.* Learner-to-Learner Interaction is positively correlated with Learner Retention in MOOCs.

Perceived effectiveness (referring to a measure of satisfaction with the learning environment), satisfaction, course organization, learning analysis, and social interaction, rather than learning evaluation, significantly influenced the continuance intention of the use of MOOCs (Yang & Sun, 2018). Institutional and course factors play a major role in the success of MOOC-based learning, and instructors and learners also act as influencing factors (Abdullah et al., 2015). MOOCs’ Content, structure, and interaction with the instructor significantly influenced Learner Retention in MOOCs (Hone & El Said, 2016). The structure of the course could significantly influence students’ retention and effect of MOOC-based learning (Kim et al., 2021). The content of course is also closely related to students’ self-directed learning effectiveness (Kim et al., 2021). Perceived effectiveness tended to be used as a variable to measure behavioral intention in online learning (Davis, 1989). Perceived Effectiveness also exerted a significant influence on Learner Retention in MOOCs (Hone & EI Said, 2016). We, therefore, proposed the following hypotheses:

*H3.* Course Content is positively correlated with Learner Retention in MOOCs.
*H4.* Course Structure is positively correlated with Learner Retention in MOOCs.
*H5.* Perceived Effectiveness is positively correlated with Learner Retention in MOOCs.

Instructors also exert a great influence on Learner Retention in MOOCs. Instructor’s coaching and prerequisites in the module element and the rate of MOOCs follow-up played the most important roles in the integration of information and communication technologies for education (Riyami et al., 2019). Maintaining users’ retention after the second stage is important to encourage learners to continue the use of MOOCs. The instructor’s presence in different forms, for example, support and feedback, could strengthen the effectiveness and Learner Retention in MOOCs (Barbera et al., 2017). Thus, we proposed the following research hypotheses:

*H6.* Instructor Support is positively correlated with Learner Retention in MOOCs.
*H7.* Instructor Feedback is positively correlated with Learner Retention in MOOCs.

### Technology

Technologies used in MOOCs are also important influencing factors. Computer self-efficacy, performance expectancy, and system information delivery quality greatly influenced the intention to use MOOCs (Fianu et al., 2018). System quality, information delivery, and technology greatly influenced the continuance intention of MOOCs users (Yang et al., 2017). Technology quality could also play an important role in MOOC-based learning achievement and student performance (Alyoussef, 2021). Thus, we proposed the following research hypotheses:

*H8.* Information Delivery is positively correlated with Learner Retention in MOOCs.
*H9.* Technology is positively correlated with Learner Retention in MOOCs.

### Social and Pedagogical Factors

It is necessary to include social and pedagogical factors when we explore influencing factors in MOOCs although social influence and effort expectancy do not exert a great influence on the use of MOOCs (Fianu et al., 2018). A framework was proposed by Azevedo and Marques (2017) on success factors for MOOCs, involving social (e.g., new experience view, timing, reputation or brand), organizational (e.g., technology), and pedagogical factors, for example, focus of subjects (referring to the focus of interesting, professional, and innovative subjects), quality resources (referring to high-quality learning resources), interactivity and peer-to-peer pedagogy, content organization and access, and timing (referring to the design of MOOCs where the schedule, and self-management of time could meet learners’ individual needs). Therefore, we proposed the following research hypotheses:

*H10.* Quality Resources are positively correlated with Learner Retention in MOOCs.
*H11.* Focus of Subjects is positively correlated with Learner Retention in MOOCs.
*H12.* Timing is positively correlated with Learner Retention in MOOCs.

MOOCs could improve learning outcomes, and both course design and active participation exerted an influence on academic achievements (Castano-Garrido et al., 2017; Yu, 2022b). Flexibility and Scaffolding for Diversity (referring to the design where adaptive contents of MOOCs are provided for learners on different levels; advanced learners can select the difficult contents, while primary learners the easy ones.) in organizing MOOCs courses could strongly influence the sustainability of MOOCs (Hjeltnes & Horgen, 2016). We, therefore, proposed the following research hypothesis:

*H13.* Flexibility and Scaffolding for Diversity is positively correlated with Learner Retention in MOOCs.

Pre-course information was considered an important factor influencing the quality of MOOCs, which was expected to declare the sort, objectives, and content of the course to potential learners before they enrolled (Alraimi et al., 2015; Creelman et al., 2014). The learners who were not satisfied with the pre-course information would possibly leave the MOOCs, while those who were satisfied would probably enroll. Pre-course information is essential as students tend to withdraw if they find it inadequate. Thus, we proposed the following research hypothesis:

*H14.* Pre-Course Information is positively correlated with Learner Retention in MOOCs.

## Research Methods

The PLS-SEM is considered an effective method to identify the cause-effect relationships among multiple variables (Hair et al., 2011). PLS-SEM has become a popular approach to multi-variable analyses in the discipline of social sciences to examine the complex relationships among numerous variables based on proposed research hypotheses (Yu, 2020; Zhonggen & Xiaozhi, 2019). This study will use SEM to examine factors that influence learner retention in MOOCs during the COVID-19 pandemic time. A mixed research method was adopted in this study to collect both qualitative and quantitative data (Trafimow, 2014).

### Participants

All the participants were undergraduate or graduate students, as well as some faculty members, in an international university. There were 147 males (60.5%) and 96 females (39.5%), ranging from 18 to 56 years old (*M* = 24.97, *SD* = 7.359). They majored in various disciplines, for example, Foreign Language, Translation, Linguistics, Chinese International Education, Chinese Language and Literature, International Politics, Journalism, International Affairs, International Relations, Finance, Macroeconomic Accounting and Analysis, Accounting, International Economy and Trade, Human Resource Management, Special Education (Speech and Hearing Science), Painting and Calligraphy, Musicology, Computer Science and Technology, Language Intelligence and Technology, Digital Media and Technology, Information Management and Information System, etc.

To filter the participants, we established the inclusion criteria. They would be included if they (a) were undergraduates, graduates, or faculty members above 18 years old; (b) had learning experiences through MOOCs; (c) had adequate English literacy and could express themselves in either English or Chinese; (d) could understand the questionnaire written in English. We obtained the participants who met the criteria (*N* = 13,578), and then randomly selected 610 of them via “Random Number Generator” in SPSS 16.0. Those who failed to meet the criteria were excluded. Three pre-undergraduate participants (one was 12 years old and the other two were 17 years old) below 18 years old were excluded because the study focused on the higher education level. We also excluded some questionnaires with incomplete or unreliable information. Finally, we obtained a total of 243 questionnaires.

We also designed a question to determine whether they had any learning experience through MOOCs to ensure that all of the participants had learning experiences through MOOCs. Those who had no MOOC-assisted learning experience were excluded. The medium of the questionnaire was English. Those failing to understand it were excluded. We obtained an independent ethical approval from each participant before the survey and interview were completed and submitted.

Since the university is an international educational institute, the participants from it came around the world, including China, Pakistan, Turkey, Malaysia, Russia, Myanmar, Indonesia, South Korea, Brazil, Bangladesh, Austria, Chile, the USA, Germany, the UK, Hungary, Egypt, Japan, India, Cambodia, Columbia, England, Norway, and Azerbaijan. They had normal literacy and psychological status based on their self-reports and researchers’ observations.

### Research Instruments

The research instrument involves a semi-structured questionnaire (Supplemental Appendix A). It contains three sections aiming to obtain demographic information, construct information, and acknowledgment. Each potential influencing factor included three items, which is followed by a five-point Likert Scale, ranging from strongly agree, agree, unknown, disagree to strongly disagree. Each response will cause five to one points respectively.

Another research instrument is a semi-structured interview (Supplemental Appendix B). This interview is composed of three sections, that is, the consent form, open-ended questions, and acknowledgment. Randomly selected 12 interviewees participated in the interviews. We selected the interviewees in a two-step process. We first numbered each of them and then produced the selected numbers using “Random Number Generator” in SPSS 16.0.

### Procedure

All the participants have experienced MOOC-based learning or teaching. Through either online or face-to-face invitations, they voluntarily participated in the study and filled in the questionnaires. Each of them was provided with a bonus after they carefully completed filling out the questionnaire. They were informed that they could complete the questionnaire at their best convenience.

**Figure 1.**
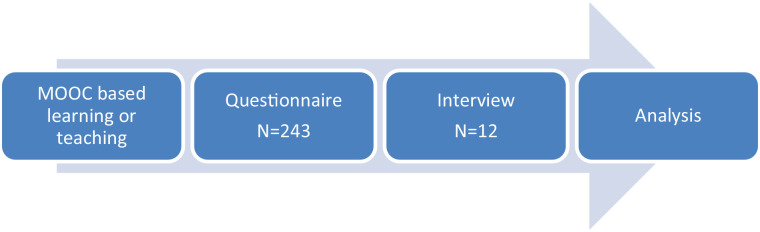
A flow chart of the research procedure.

After we obtained data from the questionnaires, we entered the data into SPSS 16.0 for further analysis. After that, to collect qualitative data, we also conducted interviews in a quiet office, where the recording equipment was provided. Twelve voluntary participants were recruited to join the face-to-face interview. The interview data were first recorded and then transcribed for further analysis (See Figure 1).

Before the interview, the interviewer obtained the interviewees’ oral consent. During the interview, the interviewer managed to create a relaxing atmosphere, in which the interviewer asked interviewees several related questions such as “Are you satisfied with MOOCs?,”“do you think it is important to maintain interactions between students and teachers in MOOCs?,”“What do you think of MOOCs’ Contents and MOOCs structures?,” and “How do you think about the timing of MOOCs?.” To encourage them to speak and obtain as much valid information as possible, they were asked to express their opinions in Chinese or English. In case they halted, the interviewer would make every effort to encourage them to continue by providing them hints or related topics, to solicit in-depth information. When they greatly deviated from the theme, we would lead them in an appropriate direction. The interviews were recorded for further analyses.

During the interview, the interviewer and interviewees followed detailed instructions. There were flexible questions that an interviewer asked the interviewees in the same way. The sequence of the question-answer is random and changeable. The interviewer probed for more answers in the same way. Some questions were structured, while others were open-ended. The interviewees could freely choose the way they responded to the questions. The interviewer provided the same time limit for all the interviewees, collected the demographic information of all the interviewees, asked for their permission before the interview, articulated the questions to make sure they were well perceived, properly asked the interviewees for more details regarding specific questions, carefully recorded the whole interview process, and finalized the interview by reminders and inquiries.

For the qualitative data retrieved from the questionnaires and interviews, we reported them through a six-step thematic analysis (Creswell & Creswell, 2017). Firstly, we familiarized ourselves with the qualitative data. We carefully listened to the audios and read the transcribed texts. We annotated the transcripts and took notes when necessary. Secondly, we coded the data by labeling the data semantically, thematically, or conceptually. For instance, we labeled the response “MOOC is a good e-learning network with lots of topics but it needs to update its information” as a requirement for “Course Content.” Thirdly, we tried to capture the important contents related to the research questions and filtered the patterned answers or meanings in the data. For instance, in the response “In general, it is more an individual than a social work, although the teacher is always willing to help with the questions and there is interaction but it is not a social methodology,” we captured the importance of “interaction” and neglect other patterned answers.

Fourthly, we reviewed the themes and rechecked if anything was missing. We also checked the quality, boundaries, ranges, quantities, or diversity of the themes. For instance, we classified the theme of the response “By doing this we can save our time to learn more about the content of the courses.” as Course Content, while we classified the theme of the response “It is important to guide students to select the course” as Pre-Course Information. Fifthly, we clarified, defined, and named the themes, and marked their distinctions and essences. Finally, we summarized the data and produced the academic report.

## Results

The results include those obtained from both the questionnaire and the interviews.

### Results From the Questionnaire

We obtained 243 questionnaires in total. Fifteen questionnaires were deleted due to the unreliable (e.g., homogeneous questions) data. This means that participants have chosen the same answer to most of the questions. We also deleted 14 questionnaires due to their incomplete information. Finally, we analyzed 243 questionnaires.

### The Test of Reliability

Before analyzing the data, we first tested the internal reliability. A reliability analysis was run to test the internal consistency. The results demonstrate that Instructor-to-Learner Interaction, Learner-to-Learner Interaction, Instructor Support, Instructor Feedback, Course Content, Course Structure, Information Delivery, Perceived Effectiveness, Quality Resources, Flexibility and Scaffolding for Diversity, Technology, Focus of Subjects, Pre-Course Information, Learner Retention, and Timing are generally internally consistent since the coefficients of them (three items for each) reached a satisfactory level (See Table 1). The total-item (*N* = 45) coefficient reached an excellent level (Cronbach, 1951).

**Table 1.** Results of the Reliability Analysis.

Value	ILI	LLI	IS	IF	CC	CS	ID	PE	QR	FSD	TCH	FS	PC	TM	LR
α	.83	.77	.70	.78	.78	.82	.82	.86	.81	.75	.71	.73	.77	.82	.84
*N*	243				Item				3			Item-total α			.96

*Note*. ILI = Instructor-to-Learner Interaction; LLI = Learner-to-Learner Interaction; CC = Course Content; CS = Course Structure; PE = Perceived Effectiveness; IS = Instructor Support; IF = Instructor Feedback; ID = Information Delivery; TCH = Technology; QR = Quality Resources; FS = The Focus of Subjects; TM = Timing; FSD = Flexibility and Scaffolding for Diversity; PC = Pre-Course Information; LR = Learner Retention.

### Pearson Correlation Analysis

Using bivariate analysis in SPSS 16.0, a Pearson Correlation Analysis was operated (See Table 2).

**Table 2.** Results of Pearson Correlation Analysis.

Item		ILI	IS	IF	LLI	CC	CS	ID	PE	LR	QR	FSD	TCH	FS	PC	TM
ILI	*r*	1														
IS	*r*	.622^**^	1													
IF	*r*	.562^**^	.711^**^	1												
LLI	*r*	.575^**^	.796^**^	.805^**^	1											
CC	*r*	.411^**^	.630^**^	.762^**^	.763^**^	1										
CS	*r*	.221^**^	.258^**^	.644^**^	.515^**^	.666^**^	1									
ID	*r*	.561^**^	.625^**^	.540^**^	.632^**^	.584^**^	.446^**^	1								
PE	*r*	.322^**^	.501^**^	.506^**^	.511^**^	.525^**^	.504^**^	.516^**^	1							
LR	*r*	.342^**^	.524^**^	.531^**^	.537^**^	.551^**^	.521^**^	.545^**^	.983^**^	1						
QR	*r*	.279^**^	.460^**^	.455^**^	.517^**^	.556^**^	.506^**^	.493^**^	.648^**^	.665^**^	1					
FSD	*r*	.353^**^	.459^**^	.522^**^	.516^**^	.625^**^	.535^**^	.450^**^	.571^**^	.580^**^	.765^**^	1				
TCH	*r*	.343^**^	.344^**^	.309^**^	.307^**^	.238^**^	.294^**^	.363^**^	.425^**^	.430^**^	.533^**^	.479^**^	1			
FS	*r*	.156^**^	.168^**^	.307^**^	.239^**^	.299^**^	.421^**^	.152^**^	.316^**^	.321^**^	.384^**^	.365^**^	.268^**^	1		
PC	*r*	.328^**^	.373^**^	.268^**^	.293^**^	.366^**^	.196^**^	.400^**^	.315^**^	.332^**^	.429^**^	.474^**^	.358^**^	.208^**^	1	
TM	*r*	.232^**^	.275^**^	.327^**^	.354^**^	.400^**^	.381^**^	.305^**^	.335^**^	.346^**^	.454^**^	.442^**^	.320^**^	.413^**^	.547^**^	1
*N*		243	243	243	243	243	243	243	243	243	243	243	243	243	243	243

*Note. r* = Pearson correlation.

** Correlation is significant at the .01 level (two-tailed).

As shown in Table 2, it is revealed that in MOOCs at the .01 level, Instructor-to-Learner Interaction, Learner-to-Learner Interaction, Course Content, Course Structure, Perceived Effectiveness, Instructor Support, Instructor Feedback, Information Delivery, Technology, Quality Resources, Focus of Subjects, Timing, Flexibility and Scaffolding for Diversity, and Pre-Course Information are positively correlated with Learner Retention. Therefore, we accepted all the proposed research hypotheses.

The partial least squares (PLS) method has been widely used for SEM analyses in a wide range of fields (Kock, 2017). WarpPLS, as a reliable tool for SEM using the PLS method, provides effective functions for researchers. It can examine nonlinear functions of latent variables in SEM and analyze multivariate coefficients of associations. It integrates reliable PLS algorithms into factor-based PLS algorithms for SEM (Kock, 2017), producing precise estimates of true composites and factors and detecting modeling errors. Therefore, we adopted WarpPLS to run the SEM analysis and test the proposed research hypotheses.

**Figure 2.**
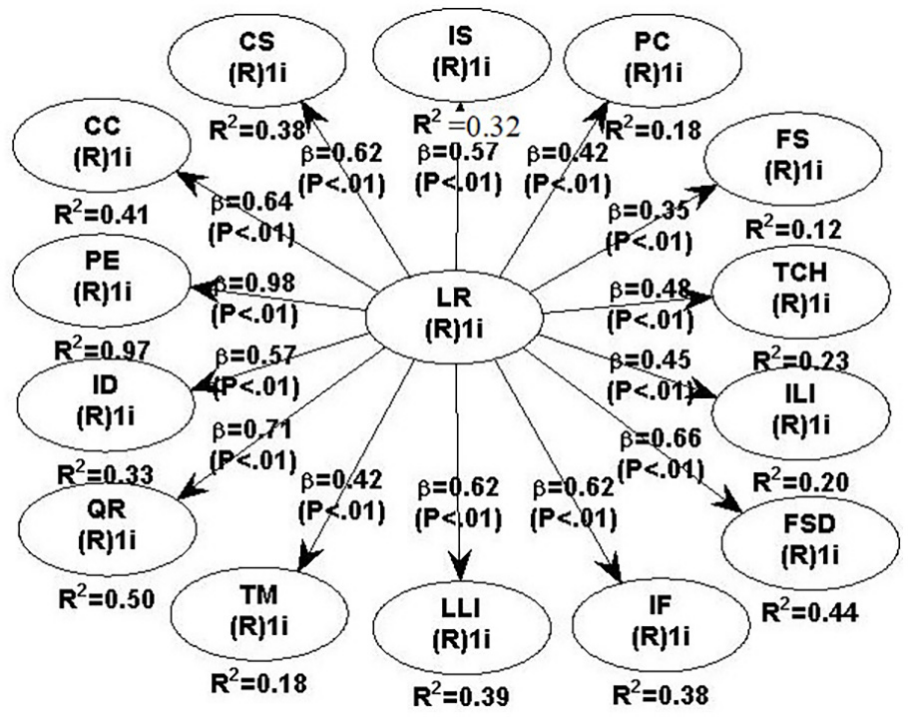
The Structural Equation Model of influencing factors.

The SEM (See Figure 2) is evidenced roughly fit due to quality indices: Average path coefficient (APC) = 0.579, *p* ≤ 5, ideally ≤ 3.3; Tenenhaus GoF (GoF) = 0.599, small ≥ 0.1, medium ≥ 0.25, large ≥ 0.36; Sympson’s paradox ratio (SPR) = 1.000, acceptable if ≥ 0.7, ideally = 1; R-squared contribution ratio (RSCR) = 1.000, acceptable if ≥ 0.9, ideally = 1; Statistical suppression ratio (SSR) = 1.000, acceptable if ≥ 0.7; Nonlinear bivariate causality direction ratio (NLBCDR) = 1.000, acceptable if ≥ 0.7.

The results of model-fit criteria and preliminary validation analysis are shown in Figure 2. Although some indicators fail to meet the goodness of model fit, most of them are well adapted, which indicates that the model has not reached the ideal state on the whole and needs to be revised, but for the acceptable level, further analysis can be made.

Figure 2 reveals the SEM of the factors influencing learner retention in MOOCs. We found that all the factors were strongly and positively correlated with learner retention in MOOCs at the .01 level. This was demonstrated by most interviewees (*N* = 9), who argued that the 15 factors played important roles in Learner Retention in MOOCs. Therefore, we accepted all the proposed research hypotheses (See Table 3 for details).

**Table 3.** Hypothesis Testing Results.

Hypotheses	Significance	Result
*H1.* ILI is positively correlated with LR in MOOCs.	β = .45, *p* < .01, *R*^2^ = .173	Accepted
*H2.* LLI is positively correlated with LR in MOOCs.	β = .62, *p* < .01, *R*^2^ = .157	Accepted
*H3.* CC is positively correlated with LR in MOOCs.	β = .64, *p* < .01, *R*^2^ = .243	Accepted
*H4.* CS is positively correlated with LR in MOOCs.	β = .62, *p* < .01, *R*^2^ = .208	Accepted
*H5.* PE is positively correlated with LR in MOOCs.	β = .98, *p* < .01, *R*^2^ = .382	Accepted
*H6.* IS is positively correlated with LR in MOOCs.	β = .57, *p* < .01, *R*^2^ = .209	Accepted
*H7.* IF is positively correlated with LR in MOOCs.	β = .62, *p* < .01, *R*^2^ = .266	Accepted
*H8.* ID is positively correlated with LR in MOOCs.	β = .57, *p* < .01, *R*2 = .261	Accepted
*H9.* TCH is positively correlated with LR in MOOCs.	β = .48, *p* < .01, *R*^2^ = .267	Accepted
*H10.* QR is positively correlated with LR in MOOCs.	β = .71, *p* < .01, *R*^2^ = .271	Accepted
*H11.* FS is positively correlated with LR in MOOCs.	β = .35, *p* < .01, *R*^2^ = .234	Accepted
*H12.* TM is positively correlated with LR in MOOCs.	β = .42, *p* < .01, *R*^2^ = .306	Accepted
*H13.* FSD is positively correlated with LR in MOOCs.	β = .66, *p* < .01, *R*^2^ = .253	Accepted
*H14.* PC is positively correlated with LR in MOOCs.	β = .42, *p* < .01, *R*^2^ = .224	Accepted

### Coefficients of Determination

The coefficient of determination (*R*^2^) aims to measure the variation proportion of the dependent variable predicted by the independent variable in a linear regression model (Zhang, 2017). The objective of the coefficient of determination (*R*^2^) in this study is to calculate the variance degree of Learner Retention predicted by the 14 independent variables in a linear regression analysis (Zhang, 2017). It is calculated as the ratio of the sum of squares of regression to the sum of squares of total deviation. Cohen (1988) reported that the coefficient will be *small* if it is about 0.01, *medium* if 0.9, *large* if 0.25.

Instructor-to-Learner Interaction can predict 17.3% Learner Retention in MOOC-based learning. Learner-to-Learner Interaction can predict 15.7% Learner Retention in MOOC-based learning. Course Content can predict 24.3% Learner Retention in MOOC-based learning. Course Structure skills can predict 20.8% Learner Retention in MOOC-based learning. Perceived Effectiveness can predict 38.2% Learner Retention in MOOC-based learning. Instructor Support can predict 20.9% Learner Retention in MOOC-based learning. Instructor Feedback can predict 26.6% Learner Retention in MOOC-based learning. Information Delivery can predict 26.1% Learner Retention in MOOC-based learning. Technology can predict 26.7% Learner Retention in MOOC-based learning. Quality Resources can predict 27.1% Learner Retention in MOOC-based learning. Focus of Subjects can predict 23.4% Learner Retention in MOOC-based learning. Timing can predict 30.6% Learner Retention in MOOC-based learning. Flexibility and Scaffolding for Diversity can predict 25.3% Learner Retention. Pre-Course Information can predict 22.4% Learner Retention in MOOC-based learning. It can be inferred that Perceived Effectiveness and Timing play the most important roles in Learner Retention in MOOC-based learning since their coefficients of determination are among the largest (>30%).

#### Results From the Interview

Results from the interviews are generally in line with those from the questionnaire. The recordings were transcribed into texts that were analyzed via WordSmith 3.0 and the thematic analysis method. Using the function of “Wordlist” of WordSmith, the top five occurring keywords are “MOOCs,”“effective,”“free,”“resources,” and “learning.” Using the function of “concordance” of WordSmith, we obtained the top five concordances, that is, “MOOCs retention,”“academic achievements,”“teaching effect,”“learning effect,” and “convenient platform.”

All of the interviewees (*N* = 12) responded that they or their children experienced MOOC-based learning either by payment or free of charge. More than half of them (*N* = 7) believed that MOOC-based learning was more effective than classroom-based learning mainly due to the flexible venue and timing. They believed peer interactions and learner-instructor interactions were very important to maintain their enthusiasm for learning via MOOCs.

The majority of interviewees (*N* = 9) reported that they preferred interactive, well-organized, and easily understood contents to difficult, disordered, and isolated ones. They also preferred pictures, videos (Yu & Gao, 2021), or cartoons to texts. Abundant and high-quality resources were eye-catching, extending students’ MOOCs retention. If instructors could respond to students’ academic issues promptly, they could learn MOOCs for much longer than without feedback. They gave priority to MOOCs that provided them with a free choice of time because they could log into MOOCs at their own convenience.

All of the interviewees (*N* = 12) preferred MOOC-based on easy and friendly technologies to difficult and unfriendly ones. In this study, friendly technologies were operationally defined as the technologies that might facilitate the learning process and could provide convenience and comfort for learners when they used the technologies. Interviewees refused to learn the difficult MOOCs. All of them did not like difficult contents. They believed that high-quality MOOCs courses could provide scaffolding skills, knowledge, and contents. They also argued that longer MOOCs retention could lead to better academic achievements.

## Discussion

Major findings in this study are at large consistent with those in previous studies (e.g. Hone & EI Said, 2016; Riyami et al., 2019; Watted & Barak, 2018; Yamba-Yugsi & Lujan-Mora, 2017). The significance of the results is that Learner Retention in MOOC-based learning is subject to various influence factors: Instructor-to-Learner Interaction, Instructor Support, Instructor Feedback, Learner-to-Learner Interaction, Course Content, Course Structure, Information Delivery, Perceived Effectiveness, Quality Resources, Flexibility and Scaffolding for Diversity, Technology, The Focus of Subjects, Pre-Course Information, and Timing. This finding ties everything back to the research questions and hypotheses.

Instructor-to-Learner Interaction is an important factor significantly influencing Learner Retention in MOOCs. As the majority of interviewees responded, they would like to use MOOCs if they could freely ask teachers questions through MOOCs. Teachers’ timely response to their questions could enhance their willingness to use MOOCs. Easy access to teachers’ guidance would possibly increase student engagement in MOOCs and thus improve their learning outcomes (Yu, Xu, et al., 2022), which would in turn positively influence Learner Retention. Forums of MOOCs are often an important tool that enables both students and teachers to interact with each other, which can be marked by the online technologies to facilitate their interactions. Traditional communicative platforms such as Twitter and Facebook are not equipped with this automatic marking function.

Learner to Learner or peer Interaction is necessary to be included to enhance Learner Retention in MOOCs. Most interviewees thought that learner interactions could encourage them to use MOOCs because they had opportunities to learn from each other via peer interactions. Peer interactions may also be able to stimulate learners to use MOOCs. Peer interactions could also have possibly created a harmonious learning atmosphere, conducive to an increase in Learner Retention (Yu, Yu, Xu, et al., 2022). Learners may feel shy faced with teachers because they worry about their possibly silly questions. Before their peers who are closer to them, they tend to feel relaxed to voice their opinions. Through the online forums in MOOCs, peer interactions could also be monitored by teachers. They are also available to peers who log into the forum. Participation in peer interaction could also contribute to their final scores through the automatic scoring system. Learner Retention may be prolonged through this interactive forum.

Instructor Support plays an important role in improving Learner Retention in MOOCs. Instructor Feedback and Perceived Effectiveness significantly influence Learner Retention in MOOCs at the .01 level. Most interviewees agreed that if teachers could contribute to peer discussions, actively help students solve difficult problems, answer students’ questions, examine students’ assignments, and attend to individual learning styles, they would possibly obtain better results through MOOC-based learning. In case of the improved effectiveness of MOOCs, they would learn via MOOCs more frequently and for longer periods. Teachers’ constructive suggestions could also encourage them to focus on MOOCs, together with longer Learner Retention. Similar to the face-to-face classroom, through MOOCs platforms, the instructor can present their image and raise questions. Learners can answer questions and feel supervised by the instructor. All learning activities can be automatically recorded by the online technologies for their final scores. This helps the instructor to check learners’ attendance and encourage their participation. It is also important to identify the specific needs of instructor support to design the MOOC-based education (An et al., 2021).

Course Content and Course Structure could also significantly improve Learner Retention in MOOCs at the .01 level. Interviewees believed that if the contents of MOOCs were challenging, interesting, and updated, they would obtain access to MOOCs. If the structure of MOOCs was well-organized, clearly displayed, and easily understood, they would also log into the portal of MOOCs and remain longer in learning through MOOCs. The preview of Course Content and Course Structure could also help learners to perceive the contents before they decide whether to participate in the MOOC-based learning. This could also increase the retention of learners in MOOCs.

Technologies used in MOOCs are significant influencing factors of Learner Retention in MOOCs at the .01 level, and Information Delivery is a significant important influencing factor. Most of the interviewees thought that mature, easy-to-use, and well-supported technologies would encourage them to learn via MOOCs. In case the interactive contents were easily communicated, and different from printed materials, they would like to try MOOCs. Future MOOCs technologies could aim at concise interfaces of MOOCs, convenient access, highly efficient workflow, rich resources, and friendly learning mechanisms.

Quality resources could also significantly improve Learner Retention in the use of MOOCs at the .01 level. Eleven interviewees believed that relevant, updated, and properly formatted learning materials could encourage them to use MOOCs. Various learning paths, scaffolding technology, and organization skill design could also encourage their retention in MOOCs. Quality resources could also be conducive to students’ learning effect. They could play an important role in the learning and instructing process, which could undoubtedly improve Learner Retention of MOOCs. Low quality-resources, despite their richness, may discourage students from continuing MOOC-based learning in case they find the resources are not beneficial to them.

The Focus of Subjects and Pre-Course Information were also important factors that could greatly influence Learner Retention in MOOCs. A pre-course survey may be beneficial to the collection of per-course information (Moore & Wang, 2021). Most interviewees held that interesting, professional, and innovative subjects could improve the use of MOOCs. If any information about the objective of MOOCs, contents, and operations could be displayed before students enroll, they could also make a wise decision on whether to attend MOOCs. On the contrary, inadequate pre-course information and a weak focus on subjects could discourage them to learn through MOOCs. Students may be attracted by the wonderful Focus of Subjects and Pre-Course Information and then make every effort to engage in learning. They may also abandon the dull Focus of Subjects and Pre-Course Information and divert to other learning contents.

Timing is an essential factor that exerts a great influence on Learner Retention in MOOCs at the .01 level. Most interviewees (*N* = 7) believed that if the schedule and management of MOOCs met students’ individual preferences, they would more likely learn via it. They also reported that shorter MOOCs were more engaging than longer ones. Appropriate timing could positively influence Learner Retention. Instructors should attempt to design short-length videos rather than long-length ones because the form could be more attractive and more effective than the latter. Learners seem unable to concentrate on video watching for a long time. Similarly, texts, lecture notes, lecture slides, and audios should also be short enough to catch learners’ attention before they lose interest.

It is noteworthy that both graduates and undergraduates may have enrolled on MOOCs for different purposes and learning goals. The former might aim at obtaining research ideas and resources, while the latter might aim to acquire new knowledge. Undergraduates’ learning goals might lie in completion of knowledge acquisition, while postgraduates might pursue in-depth perceptions of knowledge. The different purposes and learning goals might exert a great influence on learner retention in MOOCs. Researchers might pay enough attention to this and designed different styles of MOOCs accordingly.

## Conclusion

This concluding part will involve major findings, limitations, and future research directions.

### Major Findings

This study mainly contributes to a model where multiple factors that influence Learner Retention in MOOCs are included. This enriches the literature by connecting the missing link and expanding the known research. It is also beneficial for designers and manufacturers of MOOCs to improve the quality of the products. The data used is enough, sourcing from 243 participants from many countries and a great range of ages (from 18 to 56 years old). Age, as geographical precedence (that means different cultures), could get a piece of useful information to enrich this paper.

This study also provides the implications for future studies in terms of theory and practice. The emergence and development of MOOCs can bring about dramatic changes in education and facilitate the development of open education. Open education will prosper to make educational opportunities available to all learners. MOOCs will speed up the globalization of education. Access to education will grow across the world, coupled with changing learner demographic information and increasing needs in education. Social media and digital literacy will be growingly important due to the development of open education driven by MOOCs (Yu, 2022a). Besides, pedagogical theories of MOOCs will be developed. Researchers can also apply the findings in this study to examine the theories to reduce dropout rates and increase learner retention. They could also explore the theories to realize effective online pedagogy and organization, as well as their mechanisms to improve MOOC-based learning outcomes.

### Limitations

Although this study is rigidly designed, there are still several limitations. It is seldom possible to include all of the influencing factors of Learner Retention in MOOCs. Although this study recruited participants across the world, the sample can still be expanded to include more to reach a more convincing conclusion regarding multiple influencing factors. Due to the multiple variables, it seems hard to clarify each of them. For instance, it is hard to distinguish Course Structure from Course Content, and Instructor Support from Instructor Feedback.

### Future Research Directions

Other factors of MOOCs, for example, usability of the platform and participation in academic networks, were discussed in previous literature (Ramirez, 2014). Many other factors may exert some or significant influence on Learner Retention in MOOCs, which needs further research with interdisciplinary cooperation. MOOCs may be a useful strategy to contain COVID-19 and other pandemics that need to shift traditional face-to-face learning to online or blended learning. Future research could also concentrate on how to improve online or blended learning effectiveness by increasing engagement and stimulating learners’ interest in learning.

## Supplemental Material

sj-docx-2-sgo-10.1177_21582440231175371 – Supplemental material for Examining Factors That Influence Learner Retention in MOOCs During the COVID-19 Pandemic Time Click here for additional data file. Supplemental material, sj-docx-2-sgo-10.1177_21582440231175371 for Examining Factors That Influence Learner Retention in MOOCs During the COVID-19 Pandemic Time by Zhonggen Yu and Liheng Yu in SAGE Open

sj-xls-1-sgo-10.1177_21582440231175371 – for Examining Factors That Influence Learner Retention in MOOCs During the COVID-19 Pandemic Time Click here for additional data file. sj-xls-1-sgo-10.1177_21582440231175371 for Examining Factors That Influence Learner Retention in MOOCs During the COVID-19 Pandemic Time by Zhonggen Yu and Liheng Yu in SAGE Open This article is distributed under the terms of the Creative
Commons Attribution 4.0 License ( http://www.creativecommons.org/licenses/by/4.0/) which
permits any use, reproduction and distribution of the work without
further permission provided the original work is attributed as specified
on the SAGE and Open Access pages ( https://us.sagepub.com/en-us/nam/open-access-at-sage).
