# Supplementary material for: Examining Factors That Influence Learner Retention in MOOCs During the COVID-19 Pandemic Time
Source: Sage Open. 2023 May 30;13(2):21582440231175371. doi: 10.1177/21582440231175371 (PMC10230298; doi:10.1177/21582440231175371)
Supplement: sj-docx-2-sgo-10.1177_21582440231175371 – Supplemental material for Examining Factors That Influence Learner Retention in MOOCs During the COVID-19 Pandemic Time [file sj-docx-2-sgo-10.1177_21582440231175371.docx]

**Appendix A. A questionnaire to identify influencing factors in MOOC**

| **Constructs** | **Question items** | **References** |
| --- | --- | --- |
| Instructor to Learner Interaction (ILI) | ILI1. I felt it free to ask questions via MOOC.  ILI2. The instructor responded to my questions in a timely manner.  ILI3. The instructor was easily accessible to me. | Peltier et al., 2003 |
| Instructor Support (IS) | IS1. The instructor played an important role in learning via MOOC.  IS2. The instructor contributed to discussions in MOOC.  IS3. The instructor was actively helpful when students had problems. |  |
| Instructor Feedback (IF) | IF1. The instructor was responsive to student concerns.  IF2. The instructor provided timely feedback on assignments, exams or projects.  IF3. The instructor cared about my individual learning on MOOC. | Eom et al., 2006 |
| Learner to Learner  Interaction (LLI) | LLI1. Student interaction was an important learning component of MOOC.  LLI2. MOOC provided an opportunity to learn from other students.  LLI3. I had sufficient opportunity to interact with other students on MOOC. | Peltier et al., 2003 |
| Course Content (CC) | CC1. MOOC effectively challenged me to think.  CC2. MOOC assignments were interesting and stimulating.  CC3. MOOC was up-to-date with developments in the field. |  |
| Course Structure (CS) | CS1. The structure of the modules was well prepared and organized.  CS2. Projects/assignments were clearly explained.  CS3. I understood what was expected of me. |  |
| Information Delivery (ID) | ID1. The interactive content of MOOC was effectively communicated.  ID2. The interactive content of MOOC included information not covered in printed material of the same MOOC.  ID3. The interactive content of MOOC contributed to learning. | Peltier et al., 2003 |
| Perceived Effectiveness (PE) | PE1. I would recommend MOOC to friends/colleagues.  PE2. I have learned a lot in MOOC.  PE3. I have enjoyed taking MOOC. |  |
| Learner Retention (LR) | LR1. I have learned via MOOC for a long time.  LR2. I have done many exercises/assessments in the MOOC.  LR3. I have learned many MOOC contents. | Hone, & EI Said, 2016 |
| Quality Resources (QR) | QR1. MOOC provided relevant learning materials.  QR2. MOOC provided updated learning materials.  QR3. MOOC provided learning materials with proper online formats. | Azevedo, & Marques, 2017 |
| Flexibility and Scaffolding for Diversity (FSD) | FSD1. MOOC provided a range of possible learning paths to meet different needs.  FSD2. MOOC provided scaffolding self-organization skills.  FSD3. MOOC provided scaffolding technology skills. |  |
| Technology (TCH) | TCH1. The MOOC was supported by mature technology.  TCH2. I felt it easy to use online information and communication technologies.  TCH3. The hardware and software support was good. |  |
| Focus of Subjects (FS) | FS1. MOOC provided interesting subject.  FS2. MOOC provided professional subject.  FS3. MOOC provided innovative subject. |  |
| Pre-Course Information (PC) | PC1. Before enrolment, MOOC provided information about its objectives.  PC2. Before enrolment, MOOC provided information about its contents.  PC3. Before enrolment, MOOC provided information about the way it operates. |  |
| Timing (TM) | TM1. MOOC met users’ needs in terms of schedule.  TM2. MOOC met users’ needs in terms of duration.  TM3. MOOC met users’ needs in terms of self-management of time. |  |

**Appendix B. Questions for an interview**

1. Have you experienced any MOOC based learning?
2. Are you satisfied with MOOC?
3. Do you think it is important to maintain interactions between students and teachers in MOOC?
4. What do you think of MOOC contents and MOOC structures?
5. How do you think about the timing of MOOC?
6. What do you think of the quality of MOOC resources, instructor support and feedback?
7. What do you think of the technologies used in MOOC?
8. Do you think it important that you receive MOOC related information before you decide to join MOOC?
9. Do you think it is important for MOOC to be flexible and scaffolding in learning paths, technology and organization skills?
10. Do you think learning retention and perceived effectiveness are important for MOOC?
